# Supplementary material for: Electric-Field Quantum Sensing Exploiting a Photogenerated Charge-Transfer Triplet State in an Organic Molecule
Source: J Am Chem Soc. 2025 Dec 15;147(52):48028–34. doi: 10.1021/jacs.5c13547 (PMC12766726; doi:10.1021/jacs.5c13547)
Supplement: Supplementary file 1 [file ja5c13547_si_001.pdf]

Supporting Information: Electric-Field Quantum Sensing Exploiting a Photogenerated Charge-Transfer Triplet State in an Organic Molecule

Niccoló Fontana,[†] Mikhail V. Vaganov,[†] Gabriel Moise,[†] William K. Myers,[‡] Kun Peng,[†] Arzhang Ardavan,[*,†] and Junjie Liu[*,†,¶]

†*Department of Physics, University of Oxford, The Clarendon Laboratory, Parks Road, Oxford OX1 3PU, UK*

‡*CAESR, Inorganic Chemistry Laboratory, University of Oxford, South Parks Road, Oxford OX1 3QR, UK*

¶*School of Physical and Chemical Sciences, Queen Mary University of London, London E1 4NS, UK*

E-mail: arzhang.ardavan@physics.ox.ac.uk; junjie.liu@qmul.ac.uk

Contents

Estimation of the Spin-Electric Coupling Constant and Sensitivity 2

Extended Figures 3

Preliminary DFT Study of the Spin-electric Coupling 8

Estimation of the Spin-Electric Coupling Constant and Sensitivity

Here we outline the procedure used to estimate the spin-electric coupling (SEC) constant κ (Eq. 2) and the corresponding electric-field sensitivity of ACRSA. As discussed in the main text, the analysis assumes that the zero-field splitting parameter D in Eq. 1 is the only spin Hamiltonian term that is modulated by the external electric field.

The estimation of κ follows an iterative fitting procedure. Within this framework, κ is modelled as a Gaussian-distributed parameter, whose broadening may originate from either the applied E-field inhomogeneity or strains to the D parameter. For each trial distribution of κ, the modulation of the zero-field splitting, $\delta D(E, \theta)$, is computed using Eq. 2 for all values of θ, defined as the angle between the molecular symmetry axis (z axis in the main text) and the static magnetic field B_0. The corresponding electric-field-induced shift in resonance frequency, $\delta f(\theta)$, is then calculated as the difference between the eigenvalues of the unperturbed $\hat{\mathcal{H}}(D)$ and the perturbed $\hat{\mathcal{H}}(D + \delta D(E, \theta))$ Hamiltonians.

The resulting θ-dependent SEC oscillations, $\cos(2\pi\, \delta f(\theta)\, t_E)$, where t_E denotes the duration of the E-field pulse, are combined over all orientations to generate the total spin-echo response, with each contribution weighted according to the angular distributions reported in Fig. 3(a) for $E \parallel B_0$ and in Fig. S5 for $E \perp B_0$. This weighted summation yields the simulated SEC oscillation patterns, which are then compared with the experimental data. The value of κ is iteratively refined until the normalized squared deviation between simulation and experiment falls below a predefined convergence criterion.

The κ value reported in the main text, obtained from this fitting procedure, gives rise to the simulated SEC oscillations shown in Fig. 4(c,d), which closely reproduce the experimental data. This optimized parameter was subsequently used to determine the maximum electric-field sensitivity of the spin sensor, reported as 0.51 Hz/(V/m), defined as the ratio between the mean frequency shift δf at the Z-field position (Fig. 1(c)) for $E \parallel B_0$ and the applied electric-field amplitude.

Extended Figures

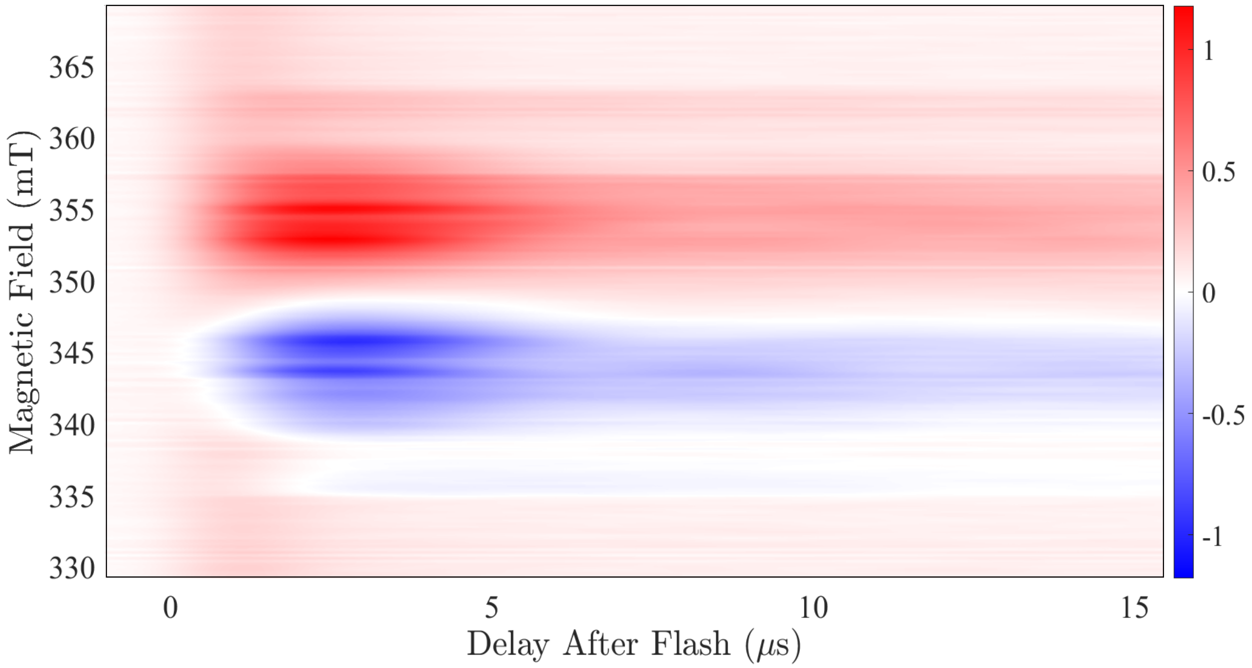

Figure S1: EPR spectrum of a 5% ACRSA-doped PMMA film measured at 20 K under 355 nm photoexcitation, shown as a function of the time after the laser pulse (known as delay after flash). The data reveal a long-lived triplet state, with the spectral features in Fig. 1(c) persisting for more than 15 μs, i.e., significantly longer than the phase memory time of the electron spin at the same temperature (~ 2.5 μs; see main text). Blue and red areas indicate the emissive and absorptive components of the spectrum, respectively.

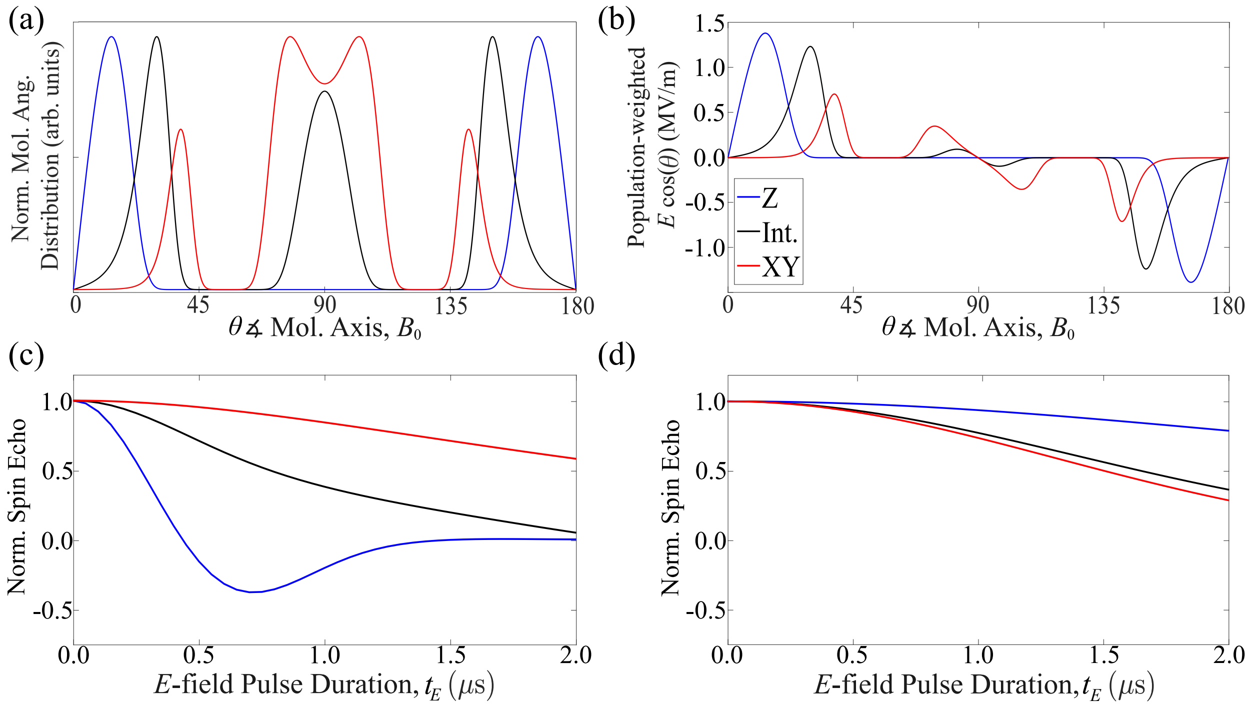

Figure S2: The simulations for (a) the angular distribution, (b) the effective electric coupling, and the electric spin-echo modulations for (c) $E \parallel B_0$ and (d) $E \perp B_0$ were repeated with a negative magnetic anisotropy D. These simulations yielded results indistinguishable from those presented in the main text, indicating that the spin-electric-coupling model is insensitive to the sign of D.

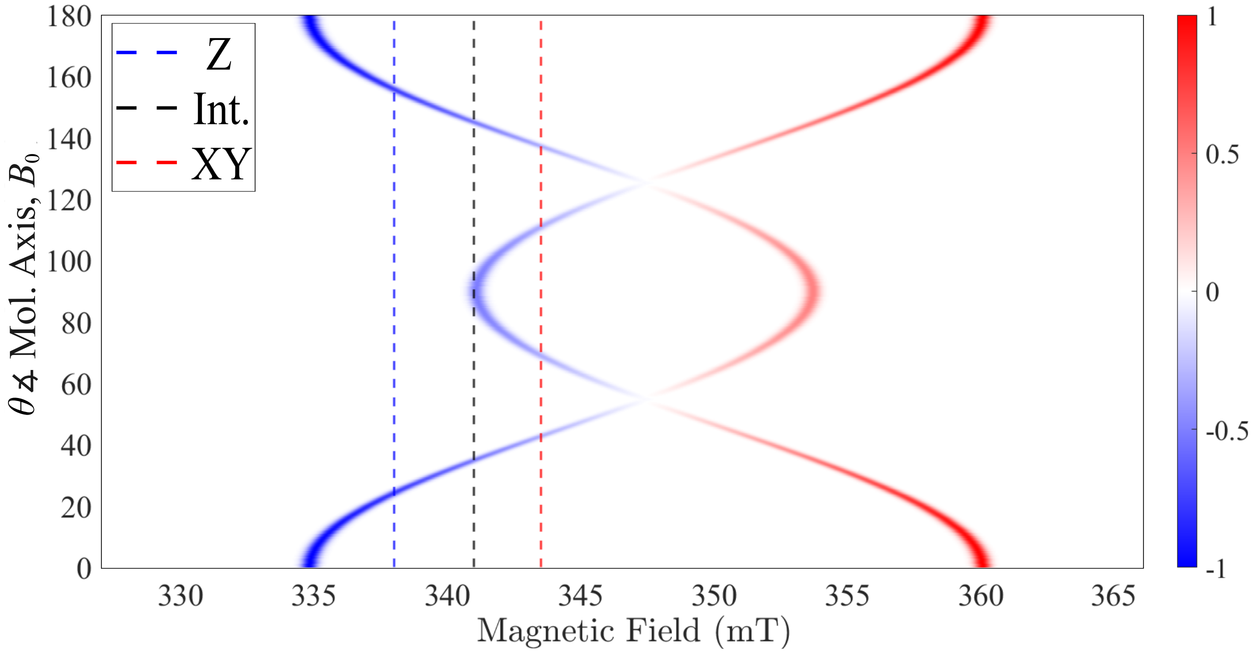

Figure S3: Simulated resonance fields for EPR emission (blue) and absorption (red) transitions as a function of the angle θ between the molecular axis and the static magnetic field B_0. The three vertical dashed lines indicate the specific field positions where we performed spin-electric coupling measurements in ACRSA. By extracting cross-sections at these field values, we obtained the simulated molecular orientation distributions shown in Fig. 3(a) in the main text. These, in turn, explain the presence of the three-peak structure observed in the angular distributions at the XY and Int. field positions.

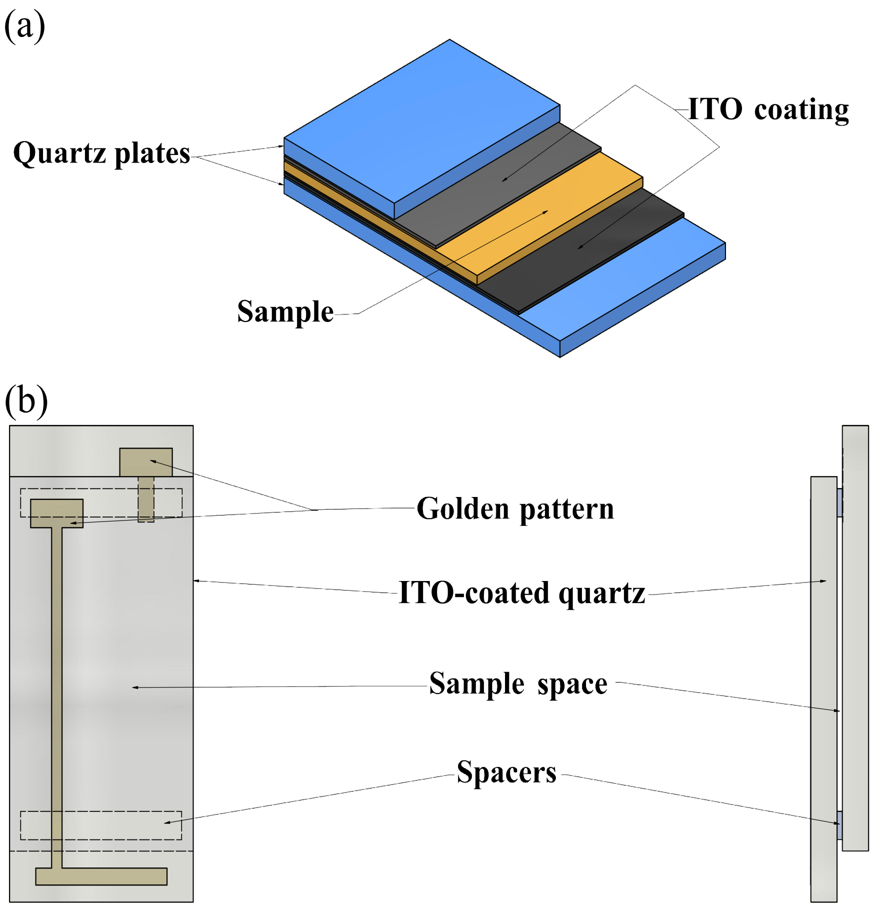

Figure S4: (a) A false-colour scheme of the E-field device used in the measurements. The DC electric-field pulse is generated by applying a voltage across a parallel-plate capacitor, whose electrodes are made from quartz coated with indium-tin oxide. These electrodes are nearly perfectly transparent to UV and microwave excitations, thus allowing both the initial laser and EPR pulses to interact with the sample. (b) A more detailed schematic of the device, illustrating the T-shaped golden patterns and the offset between the two plates. These features ensure multiple wire bondings between the device and the E-field generator. With the pulse intensity set to 300 V and the separation between the two capacitor plates roughly equal to 200 μm, an electric field of $\sim 1.5 \times 10^6$ V/m is generated at the sample site.

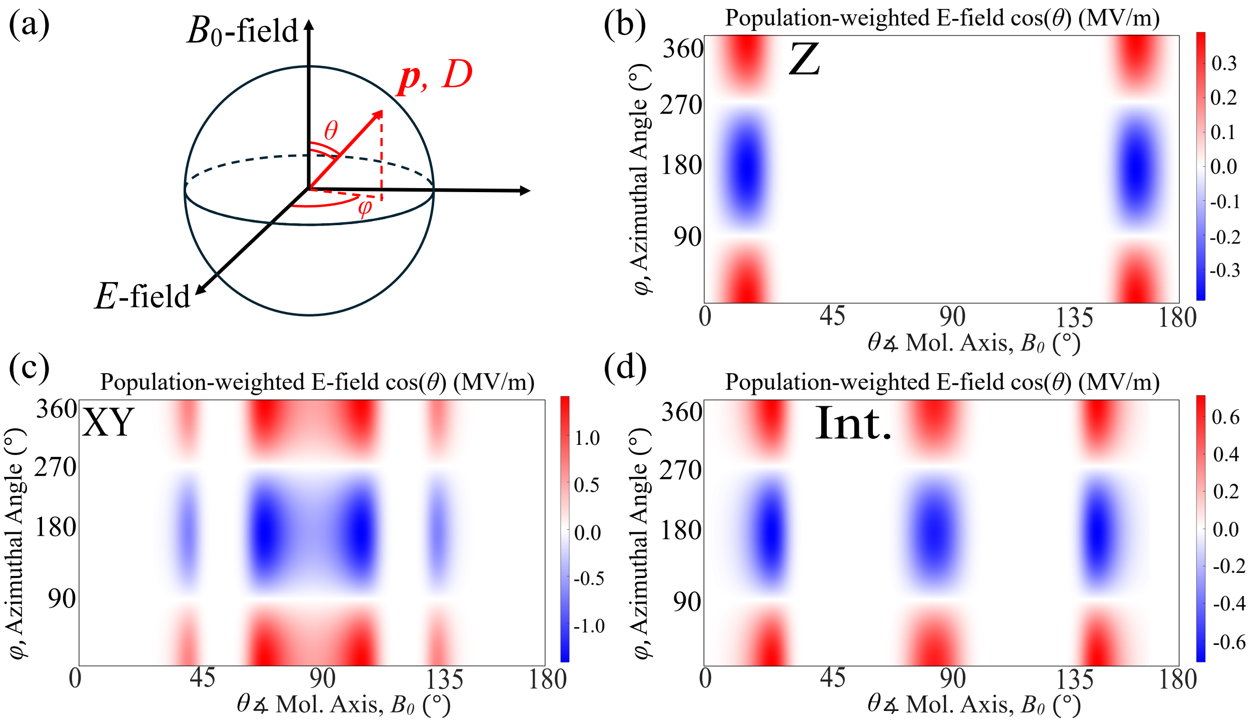

Figure S5: Schematic illustration of the molecular orientation (red arrow), with the molecular axis labelled as "$D, \mathbf{p}$", in the configuration $E \perp B_0$. In contrast to the $E \parallel B_0$ case discussed in the main text, the angle between the E-field and the molecular axis (determining the strength of the spin-electric coupling) depends on both the polar angle θ between the molecular axis and B_0, and the azimuthal angle φ between the plane spanned by $\{E, B_0\}$ and the molecular axis. (b-d) Angular dependence of the population-weighted effective $E-$field (in MV/m) for three field positions: Z (b), XY (c), and Int. (d).

Preliminary DFT Study of the Spin-electric Coupling

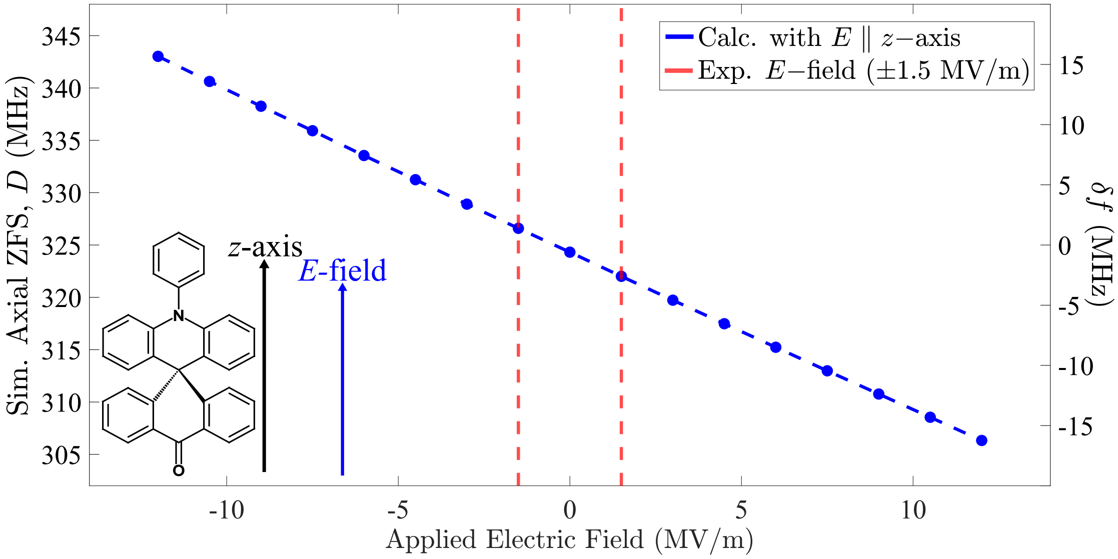

Figure S6: DFT calculations of the electric-field modulation of the zero-field splitting D (Eq. 1, main text) and the corresponding change in the EPR resonance frequency δf, performed using the ORCA package.[1] In these simulations, the electric field was applied along the molecular z-axis (see inset). The vertical red dotted lines indicate the range of electric fields explored experimentally.

The electron/hole spin densities shown in Figure 1 of the main text were obtained using the time-dependent DFT approach in ORCA.[1] The calculation employed the B3LYP functional in conjunction with the EPR-II basis[2] (optimised for the computation of EPR parameters). The magnetic–dipolar (spin–spin) contribution to the axial zero-field splitting (ZFS) D yields $D^{\mathrm{sim}} \simeq 325$ MHz, in good agreement with the measured value of 317 MHz. Building on this result, we evaluated the electric-field dependence of the axial ZFS using ORCA's dipolar `EField` functionality (see input file in Table 1). All calculations were performed with the zero-E-field DFT optimised structure included in Table 1. The calculation result is shown in Fig. S6. The linear correlation between D and the applied electric field is consistent with the experimental results. Although promising, these results should be regarded as a preliminary analysis; a comprehensive theoretical treatment remains an open challenge, as quantitative predictions of spin–electric interactions in excited states are still relatively unexplored.

Table 1: The input ORCA file uses the `EField` function to apply a uniform electric field (in atomic units) along the z-axis of the molecule, with the results shown in Fig. S6 obtained by varying the field magnitude. To simulate the effect of the PMMA host matrix, a continuum dielectric environment is included, with the relative permittivity and refractive index taken from Refs.[3,4]

```
! B3LYP EPR-II D3BJ VERYTIGHTSCF PAL32
%scf
   EField 0.25e-5, 0, 0
end
%cpcm
   epsilon 4.5
   refrac 1.5
end
%eprnmr
   DTensor SS
   DSS UNO
end
*xyz 0 3
O -5.09111 -0.11239 0.05426
N 2.19146 0.09245 0.00730
C -3.21756 3.65381 0.01999
C -1.81011 3.73103 -0.00687
C -3.84999 2.41350 0.03186
C -1.06091 2.54347 -0.02052
C -1.58317 -3.74790 -0.06940
C -2.99275 -3.75674 -0.04399
C -3.69913 -2.55739 -0.01266
C -0.90731 -2.51707 -0.06248
C -3.79541 -0.07285 0.02645
C -3.09463 1.20318 0.01745
C -3.01837 -1.30355 -0.00535
C -1.67611 1.28212 -0.00847
C -1.59790 -1.29578 -0.03107
C 0.20175 0.08271 -3.72275
C 1.60876 0.11380 -3.66940
C -0.52990 0.05432 -2.53207
C 2.26402 0.11665 -2.44010
C 0.09907 0.05613 -1.27455
C 1.51883 0.08847 -1.22975
C -0.77218 0.01969 -0.01546
C 1.50088 0.06661 1.23419
```

```
C 0.08069 0.03276 1.25726
C -0.56630 0.00783 2.50568
C 0.14799 0.01547 3.70704
C 1.55563 0.04932 3.67494
C 2.22843 0.07474 2.45548
C 6.43746 0.19241 0.03779
C 5.76456 -1.04014 0.02625
C 4.36189 -1.07762 0.01632
C 3.63718 0.12645 0.01801
C 5.70716 1.39194 0.03995
C 4.30429 1.36331 0.03024
H -4.94097 2.32961 0.05137
H -3.81871 4.57011 0.03122
H -1.30233 4.70081 -0.01688
H 0.03476 2.59511 -0.04158
H 0.18950 -2.50234 -0.08163
H -1.01751 -4.68481 -0.09398
H -4.79319 -2.53942 0.00655
H -3.53725 -4.70785 -0.04887
H 3.35499 0.14082 -2.40439
H -0.31762 0.08011 -4.68585
H -1.62398 0.02916 -2.55956
H 2.19785 0.13565 -4.59178
H -0.38518 -0.00485 4.66237
H 2.13138 0.05602 4.60592
H -1.66057 -0.01862 2.51738
H 3.31970 0.10128 2.43524
H 3.72102 2.28919 0.03197
H 6.33111 -1.97680 0.02486
H 3.82295 -2.02994 0.00729
H 7.53183 0.21816 0.04534
H 6.22899 2.35420 0.04928
*
```

References

(1) Neese, F. et al. The ORCA quantum chemistry program package. *J. Chem. Phys.* **2020**, *152*, 224108.

(2) Sinnecker, S. et al. Spin-Spin Contributions to the Zero-Field Splitting Tensor in Organic Triplets, Carbenes and Biradicalss: A Density Functional and Ab Initio Study. *J. Phys. Chem. A* **2006**, *110*, 12267–12275.

(3) Wang, Q. et al. Contributing Factors of Dielectric Properties for Polymer Matrix Composites. *Polymers* **2023**, *15*, 590.

(4) Sultanova, N. et al. Dispersion Properties of Optical Polymers. *Acta Physica Polonica A* **2009**, *116*, 585–587.
